# Decreased CD1a + and CD83 + cells in tonsillar squamous cell carcinoma regardless of HPV status

**DOI:** 10.1590/1678-7757-2020-0702

**Published:** 2022-05-16

**Authors:** Ana Guadalupe Gama-Cuellar, Ana Lúcia Noronha Francisco, João Figueira Scarini, Fernanda Viviane Mariano, Luiz Paulo Kowalski, Rogério Gondak

**Affiliations:** 1 Universidade Federal de Santa Catarina - UFSC Departamento de Patologia Florianópolis SC Brasil Universidade Federal de Santa Catarina - UFSC, Departamento de Patologia, Florianópolis, SC, Brasil.; 2 Hospital AC Camargo São Paulo SP Brasil Hospital AC Camargo, Serviço de Cabeça e Pescoço, São Paulo, SP, Brasil.; 3 Universidade Estadual de Campinas - UNICAMP Departamento de Anatomia-Patológica Faculdade de Ciências Médicas Campinas SP Brasil Universidade Estadual de Campinas - UNICAMP, Departamento de Anatomia-Patológica, Faculdade de Ciências Médicas, Campinas, SP, Brasil.

**Keywords:** Papillomaviridae, Tonsillar neoplasms, Dendritic cells, CD83 antigen, CD1a antigen

## Abstract

Dendritic cells (DCs) are specialized antigen-presenting cells that play a critical role in the immune response against human papillomavirus (HPV) infection, and represent a therapeutic target in cancer.

**Objective:**

To identify and quantify DCs in tonsillar squamous cell carcinoma (TSCC) under the influence of HPV infection.

**Methodology:**

CD1a and CD83 antibodies were used to identify immature dendritic cells and mature dendritic cells by immunohistochemistry in 33 primary TSCC and 10 normal tonsils (NTs), respectively. For the TSCC samples, the number of DCs per area was evaluated in the intra- and peritumoral compartments. For the NTs, the quantification of DCs was evaluated in the intra- and peritonsillar compartments. HPV detection methods were determined according to the ASCO Clinical Practice Guidelines from the College of American Pathologists Guideline (2018).

**Results:**

There were fewer intratumoral CD1a+ DCs in the HPV-positive and HPV-negative TSCC groups than in the NT group (p<0.05). In the peritumoral compartment, there were fewer CD83+ DCs in the HPV-positive and HPV-negative TSCC groups than in the NT group (p<0.001). The quantification of DCs subtypes showed no statistical differences between HPV-positive and HPV-negative TSCC groups (p>0.137). Patients with HPV-positive TSCC had significantly better overall survival rate than those with HPV-negative TSCC (p=0.004).

**Conclusion:**

Tumor activity contributes to DC depletion regardless of intralesional HPV positivity. An improved prognosis has been reported in patients with HPV-positive TSCC.

## Introduction

Dendritic cells (DCs) are antigen-presenting cells responsible for specific immune responses that capture, process, and present antigens to T lymphocytes. DCs can activate other cells, such as natural killer cells, B cells, macrophages, and eosinophils as well as generate immunological tolerance.^1^ DCs are currently classified as immature (iDCs) or mature DCs (mDCs) according to their morphology and phenotype expression. In an immature state, DCs patrol tissue microenvironments and efficiently unleash various pathways when they encounter an antigen. DCs migrate to the draining lymph nodes through a maturation process that enables the presentation of pathogen-derived peptides to CD4+ and CD8+ T cells.^1,2^

Epithelial DCs play a fundamental role in antiviral immunity since T cells depend on the presentation of viral antigens by DCs. The antigen presentation process conducted by DCs generates an effective immune defence against HPV. Epidemiological studies suggest that HPV takes advantage and interferes with the cell cycle to avoid being eliminated when the immune system is compromised or deficient, resulting in persistent infections and in the development of HPV-positive malignant lesions.^3,4^

Smoking, alcohol consumption, and oncogenic HPV infection have been found to be etiological factors for tonsillar squamous cell carcinoma (TSCC).^5,6^ This relationship of TSCC with HPV has been reported to have a better survival prognosis and less risk of recurrence than HPV-negative lesions.^7^ Some investigations have shown a correlationbetween DC subsets with distinct clinical behaviors of squamous cell carcinoma of the oral cavity and larynx.^8–11^ Hence, this study aims to investigate the population of DCs in different stages of maturation in TSCC under the influence of HPV infection.

## Methodology

This study was approved, according to the Helsinki statement, by the Ethics Committees of the Antônio Prudente Foundation - Hospital do Câncer - AC Camargo (CAAE: 00741212.8.0000.5432). Thirty-three tissue samples of primary TSCC and 10 of normal tonsils (NT) after tonsillectomy were collected between January 2015 and December 2020 from the Head and Neck Cancer Service at the above institution. Medical and anatomopathological reports of all patients were reviewed. The tissue samples were fixed in 10% formalin for 24 h, and four transverse sections were obtained. All fragments were embedded in paraffin blocks and 3 μm thick histological sections were subjected to hematoxylin and eosin (H&E) staining and immunohistochemistry (IHC). For *in situ* hybridization (ISH), 5 μm thick histological sections were obtained according to the manufacturer’s recommendations.

### HPV detection

HPV detection methods for the analyzed samples were based on the American Society of Clinical Oncology (ASCO) Clinical Practice Guidelines from the College of American Pathologists Guideline^12^ (2018). Only samples with at least 70% nuclear and cytoplasmic expression values for p16 IHC, with a moderate to strong intensity, were considered as positive. ISH was performed in cases when the immunohistochemical analysis was discordant among the pathologists. For ISH, a wide spectrum probe (Y1404 Dako, Carpinteria, CA) was used for genotypes 6, 11, 16, 18, 31, 33, 35, 45, 51, and 52. A 16/18-specific probe (Y1412; Dako, Carpinteria, CA, USA) was also used. Sections from a carcinoma of the uterine cervix were used as positive controls.

### Immunohistochemistry

Immunohistochemistry reactions with primary monoclonal mouse antibodies against CD1a (010, 1:100; Dako, California, USA) and CD83 (1H4b, 1:50; Novocastra, Newcastle Upon Tyne, UK) were used to stain immature and mature DCs, respectively. Additionally, the expression of HPV was evaluated using p16^INK4a^ antibodies (EPR1473, 1:100; Abcam, Cambridge, UK). Subsequently, the slides were incubated for 60 min at room temperature with secondary antibodies using the EnVision/Dual-Link System-HRP (K4065, Dako, USA), and developed with the chromogenic substrate (K3468, DAB, California, USA). The slides were counterstained with Carazzi hematoxylin. For each reaction, negative controls were prepared by omitting the primary antibodies. Two blinded, calibrated examiners analyzed the slides.

### Dendritic cell number

Ten fields of each slide were captured at 400× magnification with a binocular optical microscope (Axiostar plus, Carl Zeiss, Oberkochen, Germany) coupled to a digital image acquisition system (A620, Canon, Lake Success, NY, USA) and a microcomputer (AOC, Miami, USA), in which the images were stored. The number of DCs per area was evaluated in the intra- and peritumoral compartments in TSCC groups. For NTs, the quantification of DCs was evaluated in the intra- and peritonsillar compartments. The number of CD1a+ and CD83+ DCs per mm^2^ of each compartment was determined using ImageJ 1.51k software (Maryland, USA).

### Statistical analysis

Values are expressed as medians with interquartile ranges. Comparison of DCs between the groups was performed using Kruskal-Wallis and Dunn’s *post-hoc* tests. The Mann-Whitney test was used to analyze the association between HPV status and the clinicopathologic findings or the number of intra-and peritumoral DCs in TSCC cases. The clinical and microscopic findings in the different groups with HPV-positive and HPV-negative TSCC were compared using the Mann-Whitney *U*, Fisher’s Exact, and Pearson chi-square tests. The log-rank test was performed to analyze the overall survival rate between HPV-positive and HPV-negative TSCC. Statistical significance was set at p<0.05. Statistical software SPSS version 23.0 was used for the analyses.

## Results

### Patient population and HPV status

All patients were followed up for a mean of 70.34 months (ranging from 2-252 months). Of the 33 TSCC cases, 26 (78.8%) were HPV-negative and seven (21.2%) were HPV-positive. There was no detection of p16^INK4a^ in the NT group. In the HPV-negative TSCC group, 24 patients were men (92.3%) and 2 were women (7.7%), with a mean age of 59.9 years (range, 45-76 years). The HPV-positive TSCC group included 5 men (71.4%) and 2 women (28.6%), with a mean age of 48.5 years (range, 43-54 years). The NT group had 6 men (60%) and 4 women (40%), with a mean age of 38.9 years (range, 15-84 years). Table 1 shows additional data.

**Table 1:** Clinical and microscopic findings in the different groups with tonsillar squamous cell carcinoma

Characteristic		HPV- TSCC (n=26)	HPV+ TSCC (n=7)	
Age, mean (range)		59.9 (45-76)	48.5 (43-54)	0.150^a^
Sex (n [%])	Male	24 (92.3)	5 (71.4)	0.190^b^
	Female	2 (7.7)	2 (28.6)	
Race (n [%])	White	25 (96.2)	7 (100)	0.788^b^
	Black	1 (3.8)	0	
Smoking history (n [%])	Smoker	25 (96.2)	6 (85.7)	0.384^b^
	Nonsmoker	1 (3.8)	1 (14.3)	
Alcohol consumption (n [%])	Yes	21 (80.8)	6 (85.7)	0.624^b^
	No	5 (19.2)	1 (14.3)	
Symptomatology (n [%])	Yes	21 (80.8)	5 (71.4)	0.469^b^
	No	5 (19.2)	2 (28.6)	
Tumor stage (n [%])	T1	7 (27)	1 (14.3)	0.748^c^
	T2	5 (19.2)	1 (14.3)	
	T3	5 (19.2)	1 (14.3)	
	T4	9 (34.6)	4 (57.1)	
Nodal stage (n [%])	N0	15 (57.7)	5 (71.4)	0.740^c^
	N1	8 (30.8)	1 (14.3)	
	N2	2 (7.7)	1 (14.3)	
	N3	1 (3.8)	0	
Staging for TSCC (n [%])	I-II	9 (34.6)	1 (14.3)	0.294^b^
	III-IV	17 (65.4)	6 (85.7)	
Morphology	Non-keratinizing SCC	7 (26.9)	4 (57.1)	0.146^b^
	Conventional SCC	19 (73.1)	3 (42.9)	
Treatment	Surgery	6 (23.1)	1 (14.3)	0.768^c^
	Surgery + Radiotherapy	9 (34.6)	2 (28.6)	
	Surgery + Radiotherapy + Chemotherapy	11 (42.3)	4 (57.1)	

TSCC, tonsillar squamous cell carcinoma. SCC, squamous cell carcinoma.

a Mann-Whitney U.

b Fisher´s Exact Test.

c Pearson Chi-Square.

### Dendritic cells

**Figure 1:**
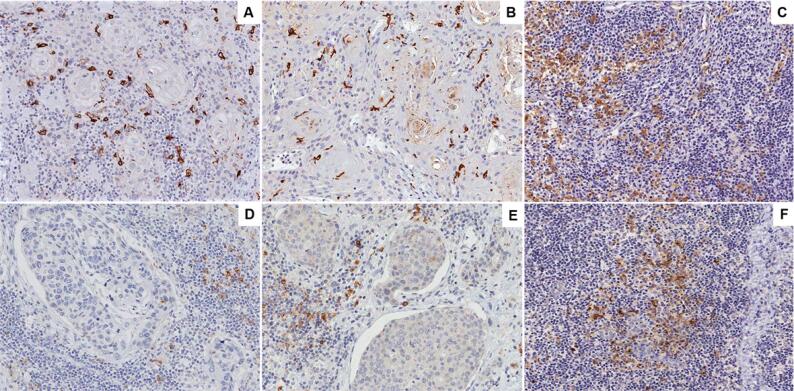
Immunohistochemical analysis of tonsillar squamous cell carcinoma (TSCC) and normal tonsil (NT). CD1a+ dendritic cells associated with HPV-positive TSCC (A) and HPV- negative TSCC (B) tumor cells. High expression of CD1a+ cells in NT sample (C). Decrease of CD83+ dendritic cells in HPV-positive TSCC (D) and HPV-negative TSSC (E) compared to NT sample (F). High magnification (x400)

CD1a+ DCs showed multiple dendritic extensions, mainly in the intratumoral compartment. In contrast, CD83+ DCs had an ovoid morphology with scarce dendritic extensions in the same compartment (Figure 1). There was no difference in the quantification of DCs within the stages of the disease (Table 2).

**Table 2:** Comparison between clinicopathologic findings and the number of intra and peritumoral dendritic cells in tonsillar squamous cell carcinoma patients (median and interquartile range)

Clinicopathologic finding		Intratumoral CD1a+ cells/mm^2^	P-value	Intratumoral CD83+ cells/mm^2^	P-value	Peritumoral CD1a+ cells/mm^2^	P-value	Peritumoral CD83+ cells/mm^2^	P-value
Staging for TSSC*	I-II	69.8 (123.8)	0.306	44.4 (60.3)	0.205	31.7 (92.0)	0.519	25.3 (19.0)	0.397
	III-IV	101.5 (152.3)		31.7 (22.2)		63.4 (38.1)		25.3 (28.5)	
Tumor stage*	T1-T2	73.0 (125.4)	0.279	41.2 (49.2)	0.371	41.2 (58.7)	0.570	25.3 (12.7)	0.742
	T3-T4	120.6 (188.8)		31.7 (26.9)		60.3 (38.1)		25.3 (28.5)	
Nodal stage*	N0	73.0 (134.9)	0.890	41.2 (49.2)	0.286	50.7 (55.5)	0.890	28.5 (23.8)	0.287
	N1-N3	101.5 (106.3)		28.5 (26.9)		60.3 (42.8)		22.2 (25.4)	
Morphology*	Non-keratinizing SCC	85.7 (228.5)	0.755	34.9 (44.4)	0.664	73.0 (90.4)	0.327	28.5 (11.1)	0.226
	Conventional SCC	88.8 (117.4)		34.9 (26.9)		47.6 (53.9)		19.0 (34.9)	

* Mann-Whitney test. TSCC, tonsillar squamous cell carcinoma. SCC, squamous cell carcinoma.

There were fewer intratumoral CD1a+ DCs in the HPV-positive and HPV-negative TSCC groups than in the NT group (*p*<0.05). A lower number of intratumoral CD83+ DCs in the HPV-positive TSCC group was observed when compared with the NT group (*p*<0.05). In the peritumoral compartment, there were fewer CD83+ DCs in both the HPV-positive and HPV-negative TSCC groups compared to in the NT group (*p*<0.001) (Table 3).

**Table 3:** Quantification of CD1a+ and CD83+ dendritic cells of all groups (median and interquartile range)

Groups	CD1a+ cells/mm^2^		CD83+ cells/mm^2^	
	Intratumoral/ Intratonsillar area	Peritumoral/ Peritonsillar area	Intratumoral/ Intratonsillar area	Peritumoral/ Peritonsillar area
HPV- TSCC (n=26)	101.58 (117.46)^a^	50.79 (53.97)	44.44 (50.79)	25.39 (25.40)^c^
HPV+ TSCC (n=7)	95.23 (269.84)^a^	76.19 (60.32)	28.57 (6.35)^a^	22.22 (23.81)^c^
Normal Tonsil (n=10)	609.52 (441.27)^b^	114.28 (85.71)	387.30 (165.08)^b^	152.38 (161.90)^d^

* Different letters in the same column indicate significant difference between the groups.

a vs

b P < 0.05;

c vs

d P < 0.01. Kruskal–Wallis and Dunn's test post-hoc. TSCC, tonsillar squamous cell carcinoma.

**Figure 2:**
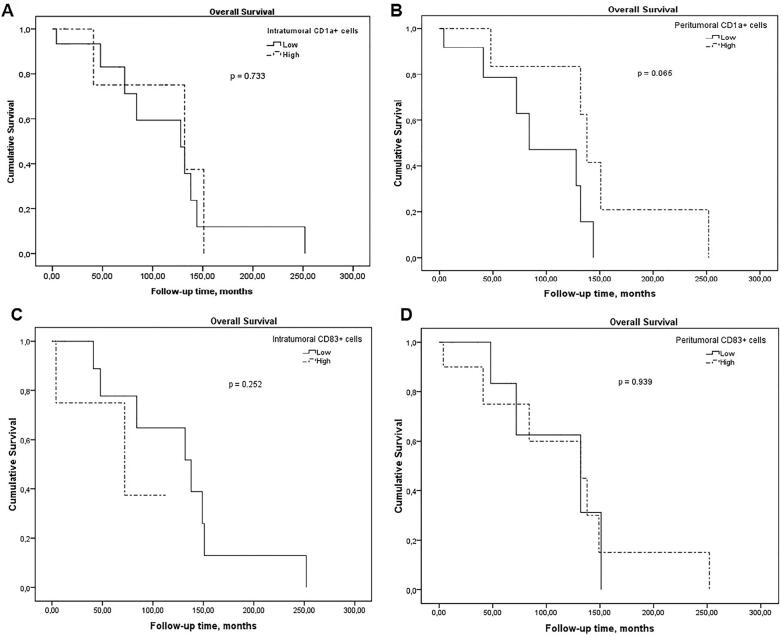
Kaplan-Meier plots of overall survival in months after initial diagnosis for patients with tonsillar squamous cell carcinoma. Stratification for (A) intratumoral CD1a+ cells; (B) peritumoral CD1a+ cells; (C) intratumoral CD83+ cells; and (D) peritumoral CD83+ cells

**Figure 3:**
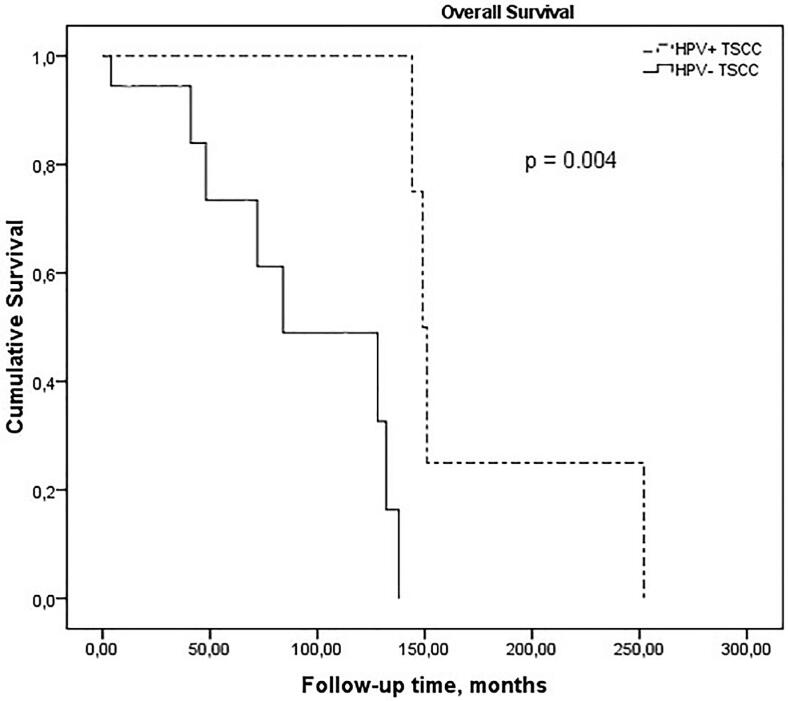
Kaplan-Meier overall survival curves according to HPV status in tonsillar squamous cell carcinoma

Survival rate of TSCC patients showed no improvement with increases or decreases in intra- or peritumoral CD1a+ and CD83+ DCs (Figure 2). However, patients with HPV-positive TSCC had significantly more favorable survival rate than patients with HPV-negative TSCC (p=0.004; Figure 3 and Table 4).

**Table 4:** Clinical outcomes for all patients with tonsillar squamous cell carcinoma

Clinical Outcome	HPV-negative TSCC (n=26) (n [%])	HPV-positive TSCC (n=7) (n [%])	P-value
Overall survival	8/19 (42)	5/5 (100)	0.004
Deceased, with disease	7 (36.7)	0	
Deceased, disease free	4 (21.1)	0	
Alive, with disease	4 (21.1)	1 (20)	
Alive, disease free	4 (21.1)	4 (80)	
Missing	7	2	

* P-value derived from the log rank test for equality of survivor functions. TSCC, tonsillar squamous cell carcinoma.

## Discussion

DC populations are an essential group of cells that act as mediators between innate and adaptive immune responses. The inhibitory or stimulatory functions of DCs depend on their subsets and maturation state. Although DCs are important in regulating an antitumor immune response, our study revealed a depletion of mature and immature DCs in TSCCs, regardless of HPV infection.

Previous studies have shown that HPV may regulate the distribution, differentiation, and function of DCs, interfering with immune surveillance.^13,14^ In this study, there was a significant decrease in CD1a+ and CD83+ DCs in HPV-positive and HPV-negative tonsillar tumors when compared with NTs. Tumor cells and the tumor microenvironment favor the release of factors that inhibit the function and maturation of DCs. Laguens, et al.^15^ (2002) showed that the densities of S100+ and CD1a+ DC in regional lymph nodes from cancer patients were significantly lower than those in the control lymph nodes. Another study^16^ showed a depletion of Langerhans cells (LC) and interdigitating cells in the epithelium of tonsils with recurrent tonsillitis, idiopathic tonsillar hyperplasia, and recurrent tonsillitis with persistent obstructive hyperplasia.

Hayati, et al.^17^ (2007) showed a lower quantity of CD1a+ DCs in cervical squamous cell carcinoma than in normal ectocervix, and these findings could be associated with tumor progression and severity. Moreover, Kindt, et al.^18^ (2016) demonstrated that a high CD1a+ LC number is associated with longer recurrence-free survival in both intratumoral and stromal compartments and longer overall survival rate in the stromal compartments of the head and neck squamous cell carcinoma specimens. We found no difference between intra- and peritumoral DC population of patients in the early and advanced stages of TSCC (Table 2). The failure in DC maturation in tumors may involve a reduced ability to stimulate an antigen-specific T-cell response due to low expression of co-stimulatory molecules.^19,20^

Gomes, et al.^10^ (2015) showed a depletion of CD1a+ and CD83+ DCs in lower lip squamous cell carcinoma when compared with the normal epithelium of patients, suggesting that an imbalance in cellular immunosurveillance would be a determining factor for the early development of lip cancer. In contrast, Costa, et al.^9^ (2016) reported a higher number of CD1a+ and CD83+ DCs in lip squamous cell carcinoma than in actinic cheilitis and in healthy labial mucosa. These divergences can be caused by other factors that contribute to the distribution and maturation of DCs, such as tobacco and ultraviolet radiation, which are strongly associated with lip cancer.

DC maturation is a continuous process that is induced and/or regulated by inflammatory cytokines. Tumor cells are known to produce several cytokines, such as TGF-β, VEGF, IL-6, and IL-10, which mediate the suppression of DC maturation and immune responses in the tumor microenvironment. Moreover, pathogen-related molecules and bacterial DNA impair DC maturation and interfere with the balance between pro- and anti-inflammatory signals in the local microenvironment.^21^

We have previously reported that conditions of immunosuppression interfere with the maturation of DCs and their functionality.^22^ CD83+ cells in cervical lymph nodes and palatine tonsils were more reduced in AIDS patients than in non-AIDS patients, and a high expression of receptors and regulatory molecules has been associated with immunosuppression. Thus, the phenotype and amount of DCs may influence tumor growth and disease progression.^23^

Some investigations have estimated that HPV causes approximately 22% of oropharyngeal cancers.^5,24^ These data corroborate our findings, in which 21.2% of tonsillar tumors were HPV positive. Previous clinical trials have shown that 50%-70% of oropharyngeal cancer cases are related to HPV.^25,26^ Variations in the proportion of HPV-associated squamous cell carcinoma (SCC) are associated with geographic differences and sexual behavior. In contrast, patients with HPV-negative head and neck tumors have a higher consumption of tobacco and alcohol.^27^

HPV-positive tumors have a better prognosis than patients with HPV-negative tumours.^6,28^ Fakhry, et al.^28^ (2008) found a 2-year overall survival rate of 95% in HPV-positive patients with SCC of the oropharynx or larynx, in contrast to the 62% survival rate of HPV-negative cases. Our findings are consistent with these studies, since we found a better survival rate in the HPV-positive TSCC (100%) group than in the HPV-negative TSCC cases (42%) (p=0.004). Several studies have pointed out that the survival improvement is due to a greater radiotherapy sensitivity or a stronger anti-tumor immune response in these tumors.^28–30^

TSCC patients with high expression of CD83+ DCs have been correlated with improved survival.^29^ However, Jardim, et al.^30^ (2018) stated that decreased peritumoral CD1a+ cells predict a worse prognosis with shorter survival time span in patients with oral SCC. Our data showed no influence on tumor infiltration by mature (CD83+) and immature (CD1a+) DCs on the survival of patients with TSCC. Variations in survival rates amongst the studies may be due to the anatomical regions affected by the tumor and the stage of the disease in which the DCs were analyzed.

Our study had some limitations. Future studies are needed with a larger sample of TSCCs associated with HPV, following the most recent ASCO Clinical Practice Guidelines from the College of American Pathologists. Furthermore, the study of other subtypes of DCs could be useful to better understand the innate and acquired immune responses against head and neck tumors.

The DC networks have been shown to contribute to the pathophysiology of several human diseases involving immunological components. Hence, developing novel immunotherapies based on DCs may induce protection and help the immune system to recognize and respond to cancer antigens.

## Conclusions

We showed that the population of mature and immature DCs in the intratumoral and peritumoral compartments of TSCC was severely decreased regardless of HPV status. Although the CD1a+ and CD83+ DC subsets showed no association with prognosis, HPV-positive tumors contributed to the greater survival of patients with TSCC.
